# Return to work of employees with low levels of education: The employers’ role and perspective

**DOI:** 10.3233/WOR-205233

**Published:** 2022-12-13

**Authors:** Nicole Hoefsmit, Inge Houkes

**Affiliations:** a Department of Work and Organizational Psychology, Faculty of Psychology, Open University, Heerlen, The Netherlands; b Department of Social Medicine, Care and Public Health Research Institute (CAPHRI), Faculty of Health, Medicine and Life Sciences, Maastricht University, Maastricht, The Netherlands

**Keywords:** Return to work, self-direction, sickness absence, sick leave, employers, employees with low levels of education

## Abstract

**BACKGROUND:**

To achieve adequate return to work (RTW) after sickness absence, Dutch legislation prescribes cooperation between absent employees and employers. Yet, we lack insight into how employees with low levels of education exercise influence over (i.e. self-direct) RTW.

**OBJECTIVE:**

This study aimed to enhance our understanding of: (A) the role that employers play in the self-direction of employees with low levels of education over their RTW; (B) how employers perceive these employees’ efforts (or lack thereof) to self-direct their own RTW; and (C) how employers understand and interpret the behaviours of these employees. Social cognitive theory served as a framework.

**METHODS:**

A qualitative study was conducted with 13 employer representatives using semi-structured interviews. Data were analysed in NVivo12 using a template approach.

**RESULTS:**

Employers tend to play a guiding, directive role in employees’ RTW. According to employers, employees generally comply with the employers’ decisions and suggestions, whether or not they have tried to realise their own preferences regarding mode and timing of RTW. Employers interpret such employee behaviours from the perspective of environmental (e.g. financial pressures to RTW) and person-related factors (e.g. sickness and RTW perceptions).

**CONCLUSIONS:**

Employers, rather than employees direct the employees’ RTW. Employers should give voice to employees and enable them to have more control over their RTW. Future research should acquire more insight in the employees’ perspective.

## 1 Background

Long-term sickness absence is disadvantageous to employees, employers and governments. Absent employees do not benefit from the generally positive role played by work in their well-being and health [1, 2], and employers and governments face high financial costs related to sickness absence and return to work (RTW) trajectories.

Given the high material and immaterial costs of sickness absence, the adequate and timely return to work of employees is important. Smeets, Hoefsmit and Houkes studied the employees’ role in their own return to work trajectory by means of a qualitative research design. They found that long-term sick-listed employees consider it very important to make their own decisions about RTW, particularly about their tasks and schedule after their RTW [3]. In another study, employees reported a need to be able to share decision making about RTW with their employers [4, 5]. Employees who manage to align their tasks and work schedules to their functional abilities and preferences during RTW [3] will likely experience a fit between their personal abilities and needs on the one hand, and the demands and supplies in their environments, i.e. person-environment (P-E) fit. Experiencing a sufficient level of fit may be beneficial to performance, well-being, job satisfaction and/or organizational commitment [6].

The above suggests that exerting a certain level of influence over their RTW is crucial for employees to achieve an early and sustainable RTW.

### 1.1 Employees with low levels of education

Employees with low levels of education have long been an understudied group in occupational health and RTW research. It is very important to pay attention to this vulnerable group as almost a third of the Dutch population aged 15 years and older has a low level of education [7]. In addition, there are educational inequalities with respect to self-rated state of health [8]. Workers with a lower level of education are at increased risk of sickness absence [9–11]. Evidence about the role of an employees’ educational level in RTW is inconsistent [12–22]. Other data show that illness or work disability is one of the reasons for non-participation of the one-third of the Dutch individuals with low levels of education (aged 25–65 years) who are inactive in the labour market. These inactive individuals are unemployed, unavailable and not searching for employment [23]. This shows that among employees with low levels of education, RTW after sickness absence is not self-evident.

Despite the recent increase in focus on the employability of low educated employees, we do not know yet whether, how or under what circumstances employees with low levels of education are supported by their employers to self-direct their own RTW. In line with Smeets et al. [3] who state that employees consider self-direction as ‘making their own decision regarding RTW’, we define absent employees’ self-direction as ‘exercising influence over RTW’. The reflections of professionals in human resources or sickness absence management will be useful in studying this topic. These professionals generally have knowledge of and extensive experience with supporting employees to resume work after sickness absence.

The aims of this study are to increase our understanding of: (A) the role that employers play in the self-direction of employees with low levels of education over their RTW; (B) how employers perceive these employees’ efforts (or lack thereof) to self-direct their own RTW; and (C) how employers understand and interpret the behaviours of these employees.

The insights from this study will be useful for employers who wish to support employees with low levels of education in achieving a sustainable RTW that meets their needs, wishes and capabilities. Such a RTW is likely to be beneficial for both employees and employers.

### 1.2 Social cognitive theory

The theoretical framework for the second aim of this study comprises parts of the social cognitive theory (SCT) [24–29]. This theory encompasses the notion of human agency. Agency embodies features such as intentionality, forethought, self-reactiveness and self-reflectiveness [29]. Self-reflection includes self-efficacy, which is an important behavioural determinant within SCT [24, 26–28, 30].

Human agency does not exist in solitude. Instead, it acts within a wide framework of influences, in which individuals both influence and are influenced by their social systems [29]. According to the SCT, human functioning is the result of reciprocal determinism [25]. This is the bi-directional interaction between each of the three core concepts: behaviour, person (cognitive, biological, and affective events) and the environment (physical structures as well as social influences) [24–26, 28, 30–32].

SCT can be applied to several social situations [25], including the RTW of employees with low levels of education. Self-direction of employees over RTW can be considered an example of human agency. In this regard, the concept of ‘behaviour’ might entail the employees’ actions in influencing their RTW. For example, employees can make suggestions to their employers about their work tasks or schedules for their RTW.

Person-related factors may underlie the behaviour of employees in RTW. These factors could be the employees’ cognitions and emotions regarding their RTW, or their medical conditions and individual characteristics such as educational level (adapted from [24, 26, 28, 30–32]). An example of such a factor is a ‘powerful others’ locus of control, i.e. ascribing control to external sources, such as medical doctors [33]. This may prevent employees from trying to influence their RTW. Also, employees who believe that they can only resume work once they have completed their medical treatment are more likely to try to postpone work resumption [4].

Environmental factors can also play a role. In the context of this study, the environment is particularly relevant with respect to the roles of stakeholders in the employees’ RTW process (adapted from [24, 26, 28, 30–32]); these stakeholders include health care professionals, colleagues and family or the government.

This study took place in the Dutch context of RTW. Dutch employers are legally obliged to pay at least 70% of the absent employees’ wages for a maximum of two years [34]. During this time, both employees and employers are obliged to abide by the Improved Gatekeeper Act. This Act states that sick-listed employees and their employers should cooperate with each other. This includes developing an action plan for the employees’ RTW before or during the eighth week of sickness absence, and meeting at least every six weeks thereafter until the employee has returned to work [35]. Although this legislation prescribes some cooperation, it does not require that employees should have to influence their own resumption of work [4, 5]. In addition, the Occupational Physician (OP) plays an advisory role for employees, their employers and the employees’ health care providers during sickness absence and RTW [36]. As such, employees and their employers are the key stakeholders who should cooperate to achieve the employees’ RTW [35].

**Fig. 1:**
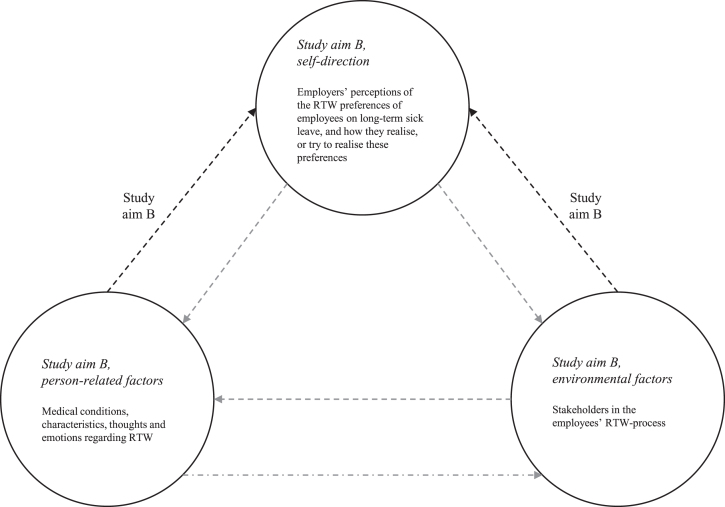
Overview of study aim B within the framework provided by the social cognitive model (adapted from [24]). *Note.* The elements displayed in black belong to the theoretical basis of this study. Also, the figure contains dashed arrows as we are interested in the participants’ perceptions of the factors that are underlying behaviour, rather than in causal relationships.

Figure 1 (adapted from [24]), shows the framework for the second study aim which consists of several core concepts of SCT and relationships between these concepts [25, 32].

## 2 Methods

### 2.1 Design

A qualitative study was conducted using semi-structured interviews with several representatives of employers. Ethics committee approval of this study was granted by the Open University of the Netherlands (correspondence 8 May 2018, registration number: U2018/03287/HVM).

### 2.2 Participants and recruitment

The interview participants were professionals in human resources or sickness absence management (further referred to as ‘employers’). They were selected because of their extensive experience with RTW processes in their organisations. Moreover, they have extensive knowledge of their organisation’s culture and sickness absence procedures, as well as of the Netherlands’ national RTW legislation. As such, they are assumed to be well able to report and reflect on the aims of this study. The inclusion criterion for employers was having experience of supporting employees with low levels of education during long-term (>6 weeks) sickness absence and RTW. In the Dutch context, persons who have completed primary education, the first three years of senior general secondary education or pre-university education, level 1 senior secondary vocational education / assistant training, or pre-vocational secondary education are usually labelled as low educated [37].

To recruit study participants we contacted approximately 40 employers. They were purposively selected to represent multiple industries in different parts of the Netherlands [38]. All employers received an email with a flyer that described the purpose of the study, the inclusion criteria, the practical procedures and information about data management (e.g. confidential treatment of data). Some of these employers were contacted by telephone. In total, 13 employers were included in this study (Table 1).

**Table 1:** Participant characteristics

Characteristics	Participants (13)
Profession	HR professional (9) / sickness absence case managers or specialists (4)
Gender	Male (1) / female (12)
Industry	Production (3), retail (3), healthcare (3), cleaning (2), transport (1), other (1)
Organisational size	≤1000 (5) />1000 (8)

Several study participants were responsible for carrying out the day-to-day management of sickness absence on behalf of their employers. Other study participants provided advice to supervisors who managed sickness absence, or were involved only in complex employee cases where supervisors did not know how to proceed.

### 2.3 Data collection

The semi-structured interviews were conducted by NH in July and August 2018. Most interviews took place at the workplaces of the study participants. Three interviews were conducted by telephone and one interview took place at a neutral location. Two study participants were employed at the same organisation, and they were interviewed simultaneously. Before the start of the interviews, the study participants gave their written informed consent. After 8–10 interviews data saturation was reached.

A topic list was used to conduct the semi-structured interviews. This list was based on the theoretical framework of this study and on a topic list used in an earlier, similar study [3]. Among other things, the list included topics concerning employee behaviours in influencing RTW, and the thoughts and emotions expressed by employees concerning their RTW processes, and the roles of stakeholders in the employees’ RTW processes.

The interviewees were explicitly encouraged to talk only about their experiences with employees with low levels of education. The interviewer asked mostly open and neutral questions. The topic list was used flexibly, allowing questioning and answering to follow a natural course. This approach also allowed the interview participants to bring up topics which they themselves considered to be relevant. The interviewer asked follow-up questions to acquire insight into these topics.

The interviews were tape-recorded. The duration of the recordings varied between 23 and 56 minutes. The average duration of the recordings was approximately 46 minutes.

### 2.4 Data analysis

The interviews were transcribed verbatim and analysed by means of NVivo 12 [39] following a template approach [40]. The analysis consisted of steps that are part of (and adapted from) the methodology described by Brooks et al. [41].

Regarding study aim B and based on the interview data, researchers developed an initial template comprising four a priori themes regarding behaviours used by employees to self-direct their RTW. These were: ‘slowing down the work resumption process’, ‘speeding up the work resumption process’, ‘aiming to keep their own job’ or ‘pursuing a new job’. For each of these behaviours, both ‘environmental factors’ and ‘person-related factors’ were added as a priori sub-themes. Please note that ‘behaviour’, ‘environment’ and ‘person’, were derived from SCT (see also Fig. 1, adapted from [24]). This template was applied to all of the interview transcripts in NVivo, and the template was modified to improve its fit with the data. Then the coded text fragments were carefully reviewed to further improve the accuracy of the names of codes/themes, and subsequently the results were described. Statements were illustrated with numbered quotes (P1-12) derived from the interview material. Please note that P2 concerns the interview with two participants. Both participants were numbered as P2.

The data were analysed by means of a ‘subtle realist’ approach [41]. Parts of an existing theory were applied to the case of employees’ sickness absence in a fairly deductive manner. As such, this study is not focused on constructing new theory which is more common in the grounded theory approach [42, 43].

## 3 Results

Below, we first describe how employers generally play both a guiding and directive role in the RTW processes of their employees (study aim A). The guiding role of employers appears to focus on making a connection with employees and supporting and protecting them, whereas the directive role concerns the behaviour of employers to speed up the RTW processes of employees and making decisions on this. We go on to describe how – according to their employers - some employees with low levels of education try to self-direct their own RTW to a certain extent, and how their efforts are understood and interpreted by their supervisors (study aim B).

### 3.1 Employers play a guiding role in the RTW processes of employees (study aim A)

Employers generally aimed to comply with the Dutch RTW legislation e.g. by making “*.. action plans. Adjustments. First-year evaluations.*” (P10) Some employers mentioned to use ICT for their administrative tasks. To illustrate: “*The supervisor reports sick using the ICT system. In this system, everything is kept up to date, so to say.*” (P2)

Employers were particularly keen to keep in touch with absent employees. “*We immediately contact absent employees.*” (P2), and some requested their employees to meet at their workplaces. “*.. we don’t want them to alienate.. * [from work, NH].” (P7) All employers made sure to contact their employees more often than required by legislation.

Most employers emphasized the importance of connecting with and supporting and protecting employees. For example, several participants mentioned the relevance of making a connection with employees through adequate communication. “*Act respectfully so people feel safe.*” (P2) In addition, multiple employers noted the importance of giving attention to employees. “*Just be curious about someone.*” (P5) Moreover, multiple interview participants suggested that employees with low levels of education are vulnerable and in need of protection. “*They are in a more dependent role, given the fast pace of society. I think this group of people needs more help with* [interpreting letters from the government, NH].” (P11) Other examples of behaviours to protect employees are to print out forms (“*..this is all you need to fill out*”, P1) or to help out with financial problems (“*Maybe we can help by saying* ‘*Okay you may repay this..*”’, P10).

It appears that not all supervisors are equally equipped to guide absent employees, and multiple employers mentioned a need for organizations to support their supervisors. “*I*’*ve noticed that many supervisors find it very though. They are trained to act like* ‘*.. a performance review and I*’*ve learnt that a performance review is a one-way conversation. So I*’*ll tell what I think of you*’..” (P5)

### 3.2 Employers play a directive role in the RTW processes of employees (study aim A)

Most employers reported their sick employees to have limited RTW possibilities with their current employers. They mentioned restrictions of individual work ability and the jobs or tasks available to them. “*They mostly are ageing people. Received no or hardly any education. Are dependent on physical work and that exactly is what made them sick.*” (P4). Yet, one employer expressed: “*..there still are quite a lot possibilities to find work that fits.*” (P11)

Employers generally play a directive role by showing behaviours that implicitly seem to stress the importance of early RTW in order to prevent loss of productivity. Nearly all interviewees mentioned involving professionals such as OPs or healthcare providers. A part of the employers was quick to consult an OP. “*..after three weeks of sickness absence, mostly two, someone* [employee] *has to visit an OP*” (P8). “*I ask the OP very explicitly* ‘*I want you to make a schedule for gradual RTW*’.” (P6) Thereby, most of them hope to speed up the RTW processes of their employees. Employers complied with the OPs advice. For example, an EL noted: “*Then you at least know the physical limitations. What is allowed and what isn*’*t*.” (P2)

In addition, employers communicated one or more of the following matters to their absent employees: the legal obligation of employees and employers to pursue RTW, the employers’ concrete decisions regarding the employees’ RTW, and their expectations of employees during sickness absence and return to work. To illustrate, one employer told an employee:“ [about a professional from a RTW agency, NH]*. This person will contact you.. to work on finding a job somewhere else.*” (P1) This quote suggests that the employer played a directive role in the RTW process.

To support early RTW, employees were allowed to make use of professional services such as “*..physiotherapist..*” (P2) An employer explained: “[There,] *often people can get treatment quickly.. It makes a big difference to us.. he doesn*’*t have to take sick leave for such a long period of time.*” (P12) This last quote illustrates that financial motives played a role.

Employers monitored and, when they considered it necessary, tried to adjust the employees’ behaviour. To illustrate, employer 4 mentioned: “[to the employee] *well, you still receive money from your employer and you cannot return the favor, well, by working. So you have to stick to other agreements and show effort. And demonstrate that you do anything possible to get well.*”

Additionally, some suggested that in the past, employees were not stimulated to resume work as much. “[Employer asks absent employees]‘*what can you still do?*’*.. this isn*’*t how things were done in the past* [at this organization].” (P11)

According to many employers, employees generally complied with their decisions and suggestions. “*They do as we say... They will follow.*”(P8) Another employer noted: “*They easily allow others to lead them.*” (P6) As the latter quote suggests, some employers thought that employees want them to take the lead on their RTW.

To nuance these findings, it must be mentioned that about half the employers tried to clarify their employees’ needs and wishes: “.. ‘*.. looking at your job, what would you be able to do? So I let them come up with solutions.*” (P6) These efforts were usually unsuccessful, for example because employees would not yet want to return to work, according to employers: “..‘*well, could you do something else?*’*.. they often say* ‘*no.. I cannot do anything*’.” (P6) Employees with low levels of education would only incidentally come up with suggestions that employers consider to be viable. “*He said* ‘*relocate me to* [a specific department, NH].’* This has become a success story.*” (P8)

Overall, our findings suggest that employers play a prominent role in shaping and directing the employees’ RTW. It appears from the data that employers consider employees to play a rather passive role in their own RTW processes.

At the same time, employers report that some employees self-direct, or try to self-direct their RTW to a certain extent (study aim B). Employers suggest that employees have preferences regarding the timing of their RTW (to return or not) and the mode of RTW (in their own job - with or without modifications - or in another job). Employees can realise, or try to realise these preferences and thereby somehow self-direct, or try to self-direct their RTW process. Nearly all interviewed employers described one or more examples of how employees self-direct their RTW (or try to).

**Fig. 2:**
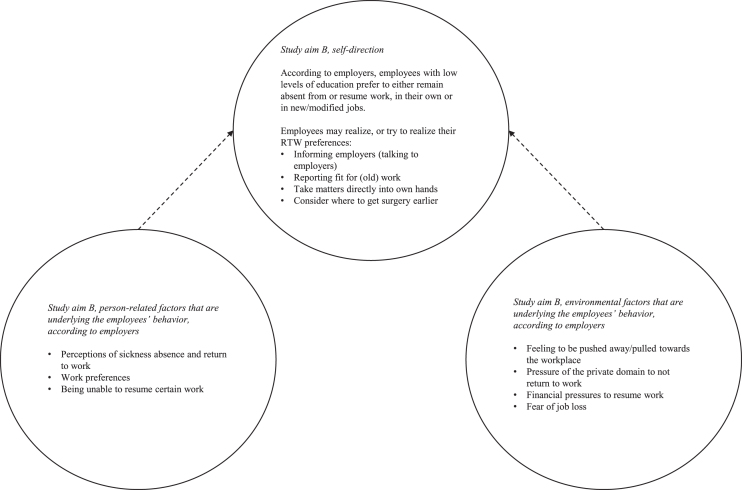
Overview of the main results regarding study aim B.

Figure 2 provides a general overview of the results regarding study aim B.

In the paragraphs below we describe how employers perceive and understand the behaviours of these employees (study aim B).

### 3.3 How some employees self-direct, or try to self-direct the timing of RTW (study aim B)

Employers described how employees may express their preference regarding the timing of their RTW by means of certain behaviours and methods of communication. All interviewees mentioned cases of absent employees who extend their sick leave, or try to extend their sick leave, while they considered this unnecessary and preferred these employees to return to work. According to slightly over half of the participants, employees told that they did not want to resume work. “.*. I don*’*t want anything, I can*’*t do anything.*” (P2). In many cases employers tended to persist though in their efforts to achieve the employees’ RTW. At the same time, multiple employers mentioned various examples of employees who make decisions (mostly successfully) beyond the employers’ influence, or employees who would try to avoid contact with employers (seemingly unsuccessfully). Some examples of the former type of employee behaviour are: “*going abroad to receive medical treatment*” (P6) and “*not sticking to the agreement* [about RTW, NH]” (P5). An example of the latter type of behaviour is “*not calling back*” (P7).

Conversely, and according to almost all interviewees, some employees would wish to return to work and try to realise this in practice. In many of these cases, employees appeared to aim to resume work quicker and/or at a more intense level than their employers preferred. Multiple interview participants mentioned cases of employees who notify them: “*I would like to work*” (P9). In a part of these cases, employees actually started to work, whereas in others employers prohibited them from working. Similarly, multiple participants mentioned employees who reported fit for work. “*I have seen this multiple times. They recover miraculously.*” (P10) According to employers, employees may report fit for work because of financial reasons for example or because their employers try to push them into a job or way of working that does not match with their preferences, i.e. they prefer to return to their own job. According to one interviewee and in exceptional cases, employees are willing to consider where they “*..can get surgery earlier*.” (P12) Please note that the statements made by employers reflect a tendency to distance themselves from their employees and to distrust their employees’ intentions regarding work resumption.

The interviewees mentioned a number of factors that they considered to play a role in the behaviours described above, i.e. perceptions of sickness and RTW (person-related factor), and financial pressures to resume work, pressure from the private domain - particularly the partner - and the feeling of being pushed away from work due to problems in the workplace or being pulled towards the workplace by satisfying social contact with colleagues (environmental factors).

#### 3.3.1 Person-related factor

***When employees are sick, they either do or do not want to resume work***

About half the interview participants suggested that absent employees tend to adopt the ‘sick role’ quite easily: “*.. sick means being incapacitated.*” (P1) According to employers, these employees do not want to work while they are sick. In addition, half of the participants thought that employees tend to follow their doctor’s advice on their capacity. Some employers suggested that their employees would prefer to take time off work instead of returning to work. An employer quoted an employee: “*In the last twenty years, I have never called in sick, and now I am sick and have to resume work immediately.*” (P2) Another employer also quoted an employee: “*It makes sense that I should still receive my salary every month, right?*” (P1) In addition, some older employees may show their frustration with the increased retirement age in the Netherlands. An employer noted: “*People are annoyed that they have to work until the age of 68 instead of 58* ...  *they started working here* [with this employer, NH] *at the age of fifteen. They simply will have to work for 45*–*50 years*.” (P1). According to this employer, these employees seem to consider it unfair that their employers push them to return to work when they are sick.

However, according to some participants, certain employees express a strong wish to work despite serious health problems: “*The people who really want* [to work, NH], *face the most problems* [health problems, NH]..” (P6) Another employer said: “*.. the people who are very seriously ill, they are the ones who show real spirit.*” (P1) According to this employer, these employees hope and aim to be able to work now or in the future.

#### 3.3.2 Environmental factors

***Employees may experience financial pressure to resume work***

Many participants mentioned various financial pressures to resume work, such as the common income reduction for employees after one year of sickness absence. “‘*Oh, I am sick and at once I get a lot less money*’*. And then they will report fit.*” (P9) According to this employer, employees who face this type of income reduction, tend to report fit for work. Please note that most Dutch employers generally pay for 70% instead of 100% of the employees’ income during the second year of sickness absence. According to some, financial pressures in the employees’ private life can push them back to work. One employer noted: “*People have considerable financial charges.* [They, NH] *want everything. And often are not able.. to think* ‘*what could I let go of* [e.g. work fewer hours, NH]*, in order to experience more balance* [in their lives, NH]?’.” (P2) Another employer mentioned the situations of divorced women who are financially dependent on their jobs.

***The employees***’ ***private domain may discourage them to work***

Multiple interviewees mentioned that the employees’ private domain may discourage them from resuming work. Employers mentioned that the opinion of the partner in particular plays a role: “*There are partners who pamper, like* ‘*no, my wife really can*’*t do anything*’*.. such an employee thinks* ‘*see, I can*’*t do anything*’.” (P4) Some employers noted that the employees’ private domain may push them into the sick role.

Many employers appeared to be conscious of and strategic about the roles of the employees’ partner or family. For example, an employer noted: “*I usually consider it pleasant when they* [absent employees] *bring their partner* [to conversations with the employer]*.. nine out of ten times you* [employer] *can get the partner on your side.*” (P4)

***Employees may feel pushed away from/pulled towards the workplace***

Half of the study participants mentioned the experiences of employees in the workplace before they called in sick. On the one hand, problems in the workplace can complicate the RTW process. To illustrate: “*.. an employee who wasn*’*t really part of the group.. he always sort of isolated himself. Well, he reported sick and right from the start, he didn*’*t feel understood.*” (P7)

On the other hand and according to some participants, satisfying social contact with colleagues can motivate employees to resume work. “*I do it* [work, NH] *for my colleagues, is what people say. If that* [social contact with colleagues, NH] *is no longer there.. (quiet). Yes, than I might as well stay at home.*” (P5) According to some, feeling concerned about colleagues can be a driver to resume work. “*.. I really have to resume work, because they are understaffed.*” (P9) One participant thought that colleagues would not make a difference in the pace of the employees’ RTW.

### 3.4 How some employees try to self-direct the mode of RTW (study aim B)

Apart from trying to continue sick leave or resume work, employers described how employees may deploy certain behaviours and methods of communication to aim for a specific mode of RTW (type of work or job). About half of the employers reported that employees may aim to resume work in their own job (even though the employers preferred work to be resumed in a different job/workplace or to work different hours). Multiple participants stated that employees expressed their unwillingness and inability to comply with the employers’ RTW plans, and some expressed indignation: “*..you don*’*t seriously think that I am going to work shifts do you?*” (P1) In a part of these cases, the employees’ efforts appeared to be successful, whereas in other cases, the employees had to comply with the employers’ intentions for their RTW, thus being given no room for self-direction. In addition, some noted that employees can decide themselves to report fit for their own job. “*He reported fit for his own job, despite the fact that the OP says* ‘*he is not suitable for that*’.” (P1)

Conversely, some employers said that employees may aim for modified work or a new job (with their current employer or elsewhere). For example, employees may tell their employers about their preference to take up new or modified work. “*..people* [employees with low levels of education, NH] *indeed say like* ‘*hey, I think I would like that and think I can do it*’.” (P12)

The interviewees mentioned to interpret the behaviours employees use to try or realise work resumption in their own jobs or new/modified jobs by two person-related factors, i.e. work preferences and being unable to resume certain work and one environmental factor, i.e. fear of job loss.

#### 3.4.1 Person-related factors

***Employees may have preferences for certain work***

Multiple interview participants mentioned employees’ personal preferences regarding the work they would like to do. “*She liked it, so she went for it.*” (P2) The interview data suggest that individual preferences may encourage employees to aim to resume their own jobs or take up a new job (mostly with their current employers).

***Employees may consider themselves unable to do certain types of work***

Some noted that employees may consider themselves unable to do certain types of work. On the one hand, employees may obstruct returning to work in a new type of work, or a modified job with their current employers. “[About employees who resume work in a new workplace, NH] *..sometimes, it goes wrong. Then you are trailing 1 nil.* [employee quote, NH] ‘*See, this is a confirmation that I cannot do it*’.” (P4)

Alternatively, and according to one interview participant (P2), when an employee has accepted his/her inability to work for his/her current employer (“*She understood that she couldn*’*t keep on doing this work.*”), avenues for exploring a different future outside of the current employer may be opened up.

#### 3.4.2 Environmental factor

***Employees may fear that they will lose employment***

Multiple interview participants said that employees may experience the threat of losing income. For example, they can experience financial uncertainty when employers start talking about finding a job elsewhere. “*..when we* [employer, NH] *aim for return to work elsewhere..* [the employee says, NH] ‘*You want me to leave*’*.. a lot of people are dependent on their incomes.*” (P2) In addition, a participant talked about employees who are absent due to sickness for almost two years: “*often.. because of the uncertainty, someone says* ‘*oh maybe I can return to my own job*’.” (P12) This employer suggests that financial uncertainty may push employees to aim for work resumption with their current employers.

## 4 Discussion

This study aimed to increase our understanding of the employers’ perspective on: (A) the role they play in the self-direction of employees with low levels of education over their RTW; (B) how employers perceive these employees’ efforts (or lack thereof) to self-direct their own RTW; and (C) how employers understand and interpret the behaviours of these employees.

The results show that employers tend to play both a guiding and directive role in employees’ RTW, leaving employees with limited decision latitude and few possibilities for self-direction in the RTW process. For example, employers usually can and do decide about the timing and mode of employees’ RTW. According to employers, employees generally complied with their decisions and suggestions, whether or not they took the opportunity to try to influence their RTW and the timing thereof. Examples of actions that employees can deploy are expressing their wishes to their employers, and reporting fit for their own job. According to employers, employees with low levels of education usually prefer, and try to remain absent from work as long as they are sick; this would sometimes be encouraged by people in their private environment. Moreover, according to employers, problems in the workplace prior to the sick leave episode can be a barrier for RTW. Yet, employers mentioned that employees with low levels of education may still return to work, or at least try to, due to financial reasons, valued contacts with colleagues and/or a wish to work despite health problems. Employers noted that these employees usually prefer to return to their own, unmodified jobs. In certain specific cases, employees appear to aim for a new or modified job, preferably with their current employers. Whether employees try to realise work resumption in their own jobs or new/modified jobs is interpreted by employers in terms of fear of losing employment, individual work preferences and the perceived ability or inability of employees to work.

### 4.1 Discussion of content

Overall, the study findings fit well within the framework provided by the SCT [24, 25, 32]. This framework allowed us to differentiate between behaviours that employees use to self-direct their RTW, and the underlying person-related and environmental factors, as mentioned by employers (see Fig. 2). The results carefully suggest that both person-related and environmental factors are more or less equally salient for understanding the employees’ efforts to self-direct their RTW.

In this study, self-direction was defined as ‘exercising influence over RTW’. This working definition covers an implicit norm about how employees should behave during sickness absence. More particularly, our definition of self-direction requires activity aimed at realising RTW, i.e. that employees take an active stance towards work resumption.

The interviewed employers seemed to agree with this norm. Our results suggest that they expect their employees with low levels of education to make an effort to resume a type of work that fits with the employees’ abilities. The implicit norm in self-direction is also in line with the Dutch Improved Gatekeeper Act. This Act assumes that absent employees and their employers will make a mutual effort to achieve the employees’ return to suitable work [35]. Moreover, this norm is consistent with the perspectives of more highly educated employees who consider it important to decide about their own RTW [3], or at least to share decision making about RTW with their employers [4, 5].

Our study results show that certain employees express a strong wish to work despite serious health problems, which suggests that these employees take an active stance towards their RTW. To these employees, work may be a welcome distraction from their health problems. In addition, our results suggest that employees have a strong tendency to resume work when faced with a threat of losing income because of their sickness absence. As such, they may resume their work to limit their financial risks. Research has shown that good job security is considered to be more important for lower educated workers than for those with a high education [44, 45]. The wages and savings of employees with low levels of education may already have been limited prior to their sickness. In fact, about a third of those with a low income are from low-income families, which implies that they are in an economically vulnerable position [46].

However, according to employers, employees with low levels of education usually aim to remain absent from work as long as they are sick. This suggests that employees with low levels of education generally would not meet the norm of taking an active stance towards RTW that self-direction entails. Our findings raise the question of why employees generally may not succeed in actively aiming for their own RTW.

About half of the interviewed employers suggested that absent employees tend to adopt the ‘sick role’ easily, suggesting that these employees lack motivation to resume work. It is plausible that employees with low levels of education actually have limited abilities to work while they are sick. In fact, employees with low levels of education are typically skilled for blue-collar jobs that are characterised by relatively high physical work demands [47]. Physical work in itself can be a bottleneck for job preservation [23], as employees may have limited control over heavy demands [48, 49]. Moreover, employees may suffer from physical health complaints such as lower back or neck pain [50] and an overall lack of occupational physical fitness [51], which may complicate return to work. Work modification may be a solution, but our results suggest that some employees consider themselves unable to perform well in modified or new jobs.

Other research, among lower educated older workers, also shows that they are less confident in their ability to switch jobs [52]. Indeed, our findings show that when employees aim to resume work, employees with low levels of education may prefer their own jobs. They appear to hang on to what is known and familiar. Yet, in their old, unmodified jobs, employees may not always experience an optimal fit between their RTW (e.g. tasks) and their own abilities and needs, i.e., a sub-optimal P-E fit. More generally, P-E fit fosters positive outcomes such as job performance and job satisfaction [6]. In short, employees may face a lack of abilities to achieve RTW (to modified work) while they are sick, which complicates both their self-direction and P-E fit with respect to RTW.

In addition, employees may lack the knowledge and skills to take self-direction with respect to their RTW. Multiple employers suggested that employees with low levels of education are vulnerable and in need of protection. For example, employees would be unable to interpret letters from the government with respect to their work ability. As our interview data suggest, employees may be happy to rely on employers to take the lead over their RTW.

Yet, employees may also be discouraged to take an active approach to RTW, because of their employers’ behaviour. The interviewed employers considered themselves to be in a better position than employees to make decisions regarding RTW. They had extensive knowledge of, and experience with, RTW legislation. Moreover, they were well acquainted with possibilities for work modifications and both internal and external job mobility. Our results also show that employers consciously steer employees towards early RTW. Employers take employees by the hand, guiding them step by step, and make decisions about RTW themselves. Although these employers aimed to ‘do what is best for the employees’, they seem to use their hierarchical positions of power over employees to support them in a somewhat paternalistic and directive way. This leaves employees with limited options to take an active stance towards work resumption, which complicates their self-direction. Moreover, the employers’ behaviour may contribute to a sense of passiveness or helplessness in employees. More generally, individuals with a low socioeconomic status may feel they have limited control. As such, they may experience a general lack of grip on their environments and circumstances [53]. A lack of control may be exacerbated by their lack of self-direction in RTW.

## 5 Conclusion

Employers considered employees to play quite a passive role in sickness absence and RTW. This may be a reaction to the employees’ lack of abilities to achieve RTW while they are sick, their lack of knowledge and skills to take self-direction; and/or a reaction to the employers’ somewhat paternalistic and directive behaviour. Our findings show a tendency of employers to distance themselves from their employees and to distrust their employees’ intentions regarding work resumption. This reflects ‘us-them (in-group/out-group) thinking’ [54] and organizational cynicism [55], which may be bottlenecks for employers to fully understand the needs of absent employees and to provide adequate support in the return to work process.

Our study adds to the literature. Existing evidence about the role of the employees’ educational level in RTW is mainly quantitative and inconclusive [12–22]. Our qualitative study provides some in-depth insights into the RTW process of employees with low levels of education. The study findings suggest that these employees may exercise limited control over their RTW, and receive little adequate support from employers. Our study findings also add to the body of knowledge on programs to facilitate return to work (see, for example [56, 57]), as well as research on factors that play a role in RTW (see, for example [58, 59]).

### 5.1 Limitations

To ensure adequate methodological quality of this study, we purposely selected the study participants from multiple industries and from different areas of the Netherlands. Interviews were conducted using a topic list that matched with our theoretical framework. Moreover, we conducted additional interviews until data saturation was achieved. Both authors were involved in the process of data analysis.

This study also has several limitations. First, we were only able to interview employers and not employees. We did not succeed in recruiting employees with the help of HR professionals, occupational physicians and psychologists, despite intensive efforts. At the time of the data collection, the Personal Data Act was introduced. Human resources professionals in particular were afraid of violating this act somehow. Moreover, healthcare professionals lacked knowledge of the educational level of their patients or did not know an employee who would be eligible to participate in our study. As a consequence, our data allowed us to study the employers’ perspective only, which is likely to differ from that of employees. For example, many employers suggested that employees generally complied with their decisions and suggestions. However, it is likely that employees would describe themselves as taking a more active stance on their own RTW. In addition, we did not take the perspectives of OPs and supervisors into account in this study. They usually play important roles in the timing and mode of the employees’ RTW as well.

Second, we interviewed employers about employees with low levels of education; i.e. those employees who have completed primary education, the first three years of senior general secondary education or pre-university education, level 1 senior secondary vocational education / assistant training, or pre-vocational secondary education [37]. The RTW process of an employee who has only completed primary education may differ from that of an employee who has completed level 1 vocational education. Yet, our data do not allow variations within the group of employees with low levels of education to be studied.

Third, although we interviewed employers in many occupational sectors, we were unable to interview employers in the construction sector. Therefore, the study results are not representative of this sector.

Despite these limitations, our study results seem to provide the reader with a fairly representative overview of the Dutch employers’ perspective on the self-direction of sick-listed employees with low levels of education.

### 5.2 Recommendations for research and practice

Future research is necessary to acquire in-depth insight into employee RTW preferences and need for support. There is a need for qualitative studies of the absent employees’ perspectives on their RTW. These could be studies based on semi-structured interviews or on focus groups. Multiple groups of employees with low levels of education can be studied separately, e.g. with different levels of low education, employed in different occupational sectors. In addition to exploration of the perspectives of employees, more insight is needed into the roles in and perspectives of OPs and supervisors on the self-direction of sick-listed employees with low levels of education. Such knowledge can inform coordinated professional efforts of OPs and supervisors that enable these employees to self-direct their RTW.

Researchers, employees with low levels of education and employers need to work together in action research to bring about and evaluate change [60]. Our study results suggest that employees with low levels of education are vulnerable during sickness absence, as they experience difficulties in exercising influence over their own RTW. It is important to give more voice to employees with low levels of education and to enable them to have some level of control over their RTW processes. Therefore, employers should be mindful not to impose their own norms on their employees with low levels of education. Instead, employers should initiate a dialogue (adapted from [61]) with absent employees in which they are aware of their own biases towards employees. In this dialogue, employers could make an inventory of how employees with low levels of education experience their sickness absence and RTW, and of the support that this group needs. Moreover, they should tell employees with low levels of education about their legal position. In addition, employers should, to some extent, refrain from using their power (adapted from [62]). Such an approach may enable employees’ perception of their control over their RTW processes. If employees feel they are in in control of their return to work to some extent, they will likely feel enabled to self-direct their own RTW processes.

Lastly, governmental and employer policy makers could play a role in developing legislation and organisational policies to ensure that employees with low levels of education can influence in their own RTW.
